# Supplementary material for: Evaluation of the Street Canyon Level Air Pollution Distribution Pattern in a Typical City Block in Baoding, China
Source: Int J Environ Res Public Health. 2022 Aug 22;19(16):10432. doi: 10.3390/ijerph191610432 (PMC9408171; doi:10.3390/ijerph191610432)
Supplement: Supplementary file 1 [file ijerph-19-10432-s001.zip › ijerph-1856876-supplementary.pdf]

Evaluation of the street canyon level air pollution distribution pattern in a typical city block in Baoding, China

Supporting Information

Jingcheng Zhou[1], Songlin Xiang[1], Yizhou Zhang[1], Yuqing Wang[1], Wendong Ge[1], Junfeng Liu[1*], Jianying Hu[1], Yi Wan[1], Xuejun Wang[1], Ying Liu[2], Jianmin Ma[1], Xilong Wang[1], Shu Tao[1]

1. Laboratory for Earth Surface Processes, College of Urban and Environmental Sciences, Peking University, Beijing 100871, China.
2. School of Statistics, University of International Business and Economics, Beijing, 100029, China

*Correspondence to: Junfeng Liu (E-mail: jfliu@pku.edu.cn)

S1. Study Area

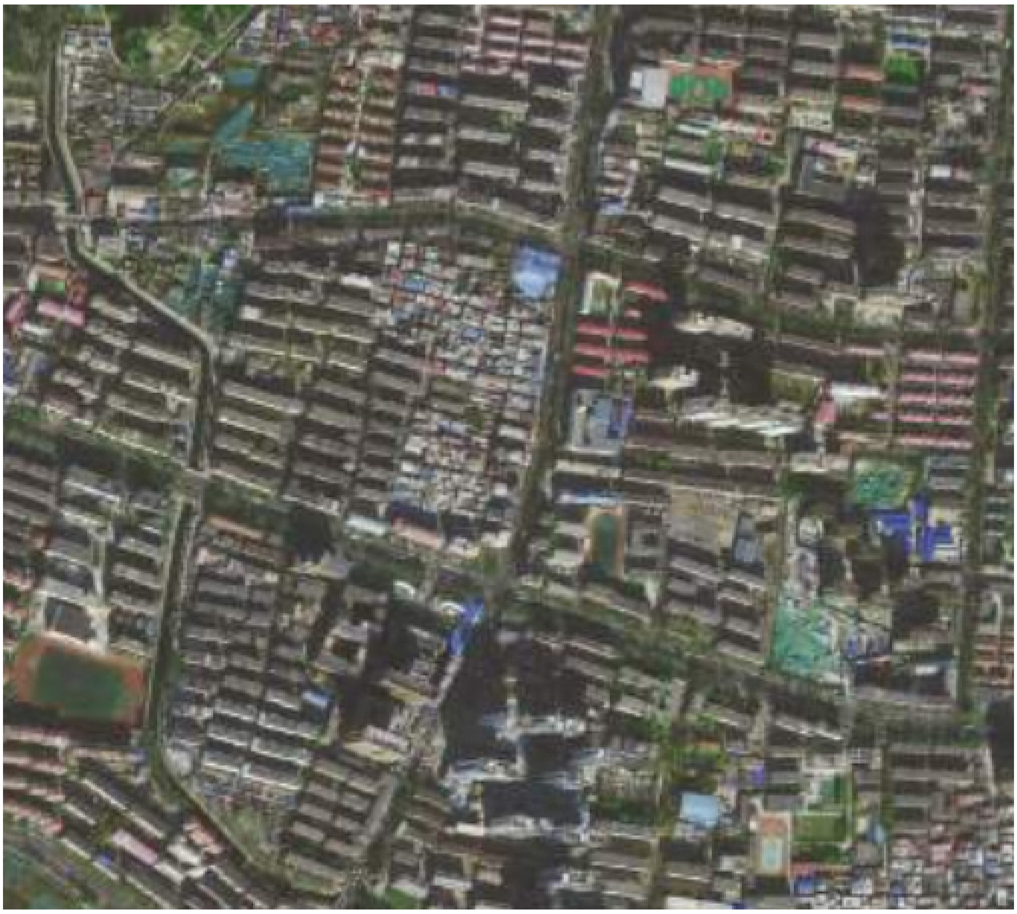

Fig. S1 Satellite map of the study area, a 1km × 1km × 200m city block in the downtown area of Baoding, China.

S2. Definition of the influencing parameters in the MLR model[1, 2]

(1) Emission: The emission is calculated with the traffic numbers, multiplied with an emission factor for different pollutants. The figure shows the emission values of NO_x.

(2) Building coverage ratio (BCR): the ratio of built-up area and site area.

$$\mathrm{BCR} = \frac{S_{build}}{S_{total}} \tag{1}$$

Where S_{build} is the building footprint and S_{total} is the total area.

(3) Asymmetric ratio: the ratio of the height on the two sides of the street canyon, representing the asymmetry of the street canyon.

$$\text{Asymmetric ratio} = \frac{H_1}{H_2} \tag{2}$$

Where H_1 and H_2 are the average building height on the two sides of the street canyon, respectively. For calculation, the numbers are adjusted to be within 0-1.

(4) Aspect ratio: the ratio of the height to width of the street canyon, representing if the street canyon is deep and narrow.

$$\text{Aspect ratio} = \ln(1 + \frac{H}{W}) \tag{3}$$

Where H is the average height of the buildings on both sides of the canyon and W is the width of the canyon.

(5) Rugosity: the mean height of the urban canopy.

$$\text{Rugosity} = \frac{\sum_{\text{built}} A_i h_i}{\sum_{\text{built}} A_i + \sum_{\text{nonbuilt}} A_j} \tag{4}$$

Where A_i is the footprint area of building i, h_i is the height of the building i, and A_j is the area of non-built element j.

(6) Porosity: the ratio of the open volume to the total volume of the urban fabric.

$$\text{Porosity} = \frac{\sum_{\text{open spaces}} V_i}{\sum_{open\ spaces} V_i + \sum_{built} V_j} \tag{7}$$

Where V_i is the canopy volume above the open space i, and V_j is the volume of the built volume j.

(7) Occlusivity: the built to unbuilt perimeter against height. It is calculated by way of a series of horizontal cuts of the urban fabric.

$$\text{Occlusivity} = \frac{1}{N_{Horizcuts}} \sum_{N_{Horizcuts}} \frac{P_{built}}{P_{unbuilt}} \tag{8}$$

Where $N_{Horizcuts}$ is the number of horizontal cuts, P_{built} is the built perimeter for the current cross section, and $P_{unbuilt}$ is the unbuilt perimeter for the current cross section. In the

study, the horizontal cuts are chosen per 3m.

(8) Distance from the main road:

This parameter is defined as the shortest straight-line distance from the main road (i.e. the roads with traffic emissions).

The multicollinearity test results of the urban morphological parameters are shown in Table S1 (The results are from a multilinear correlation using the software SPSS20, but only VIF values are shown in the table). No VIF values exceed 10, which means that no severe multicollinearity exists between the parameters [3].

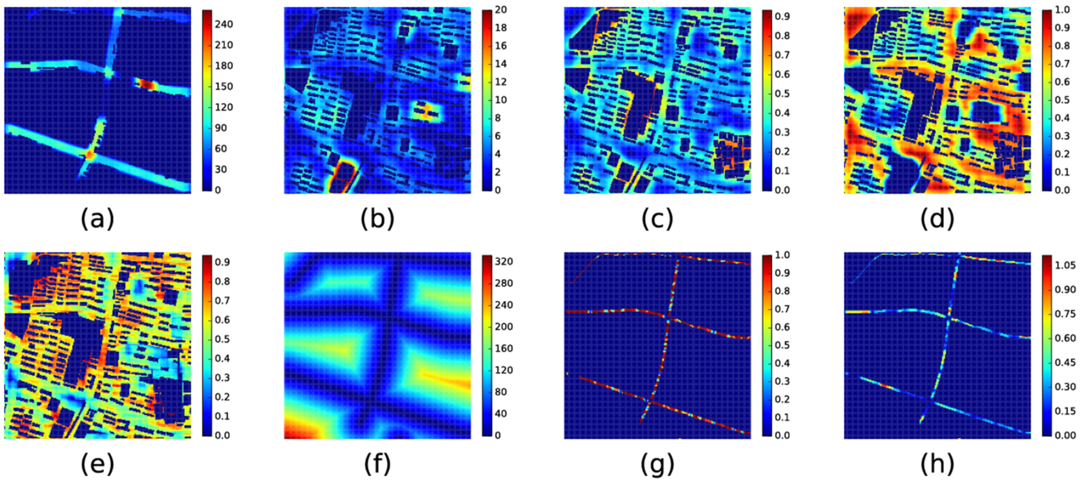

Fig.S2 Spatial patterns of (a) emissions and these morphological parameters including: (b) rugosity; (c) BCR; (d) porosity; (e) occlusivity; (f) distance from the main road; (g) asymmetry ratio; (h) aspect ratio. The parameters of the porosity, asymmetry ratio and aspect ratio are related to the features of the street canyons, so they exhibit values only along the roads.

Table S1 Multicollinearity test of the selected urban morphological parameters

Parameter	VIF
Occlusivity	5.784
Rugosity	5.768
DR	1.123
Aspect Ratio	2.354
Asymmetry	1.053
Porosity	2.357
BCR	1.068

S3. Roadside monitoring data and model validation

Fig. S3 shows the location of the study area, the two monitoring sites (marked as M.S.1 and M.S.2) and the two national sites (marked as N.S.1 and N.S.2). The scatter plot of the hourly pollution data (model results V.S. the data from monitoring site 2) is attached as Fig. S4.

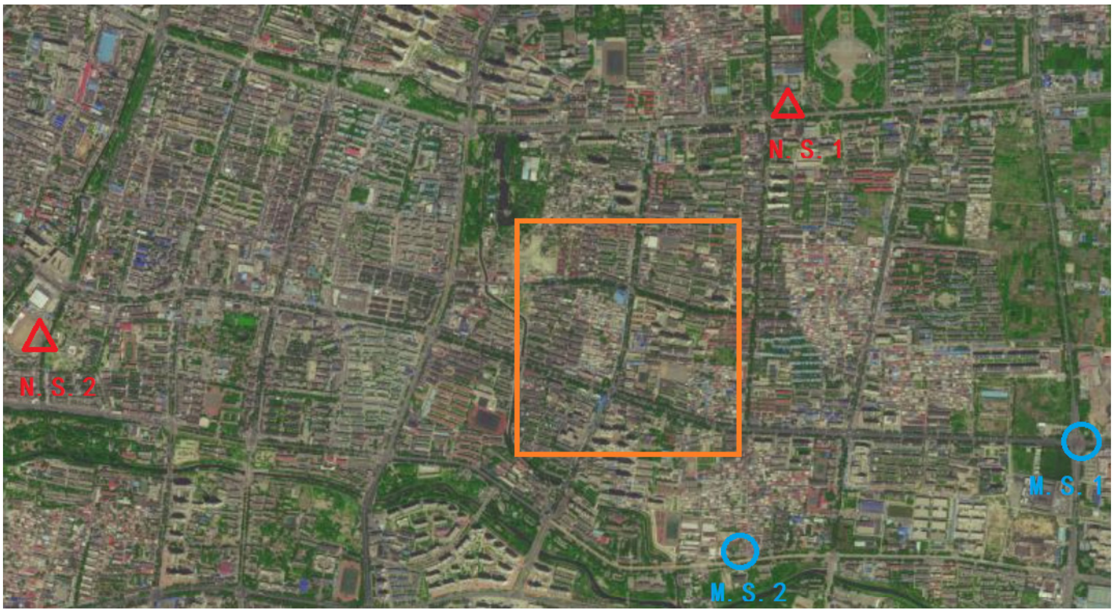

Fig. S3 The position of the study area (the orange rectangle), the national sites (the two red triangles) and the monitoring sites for model validation (the two blue circles). Note that data from the national sites are used only for a calibration of the WRF-Chem results.

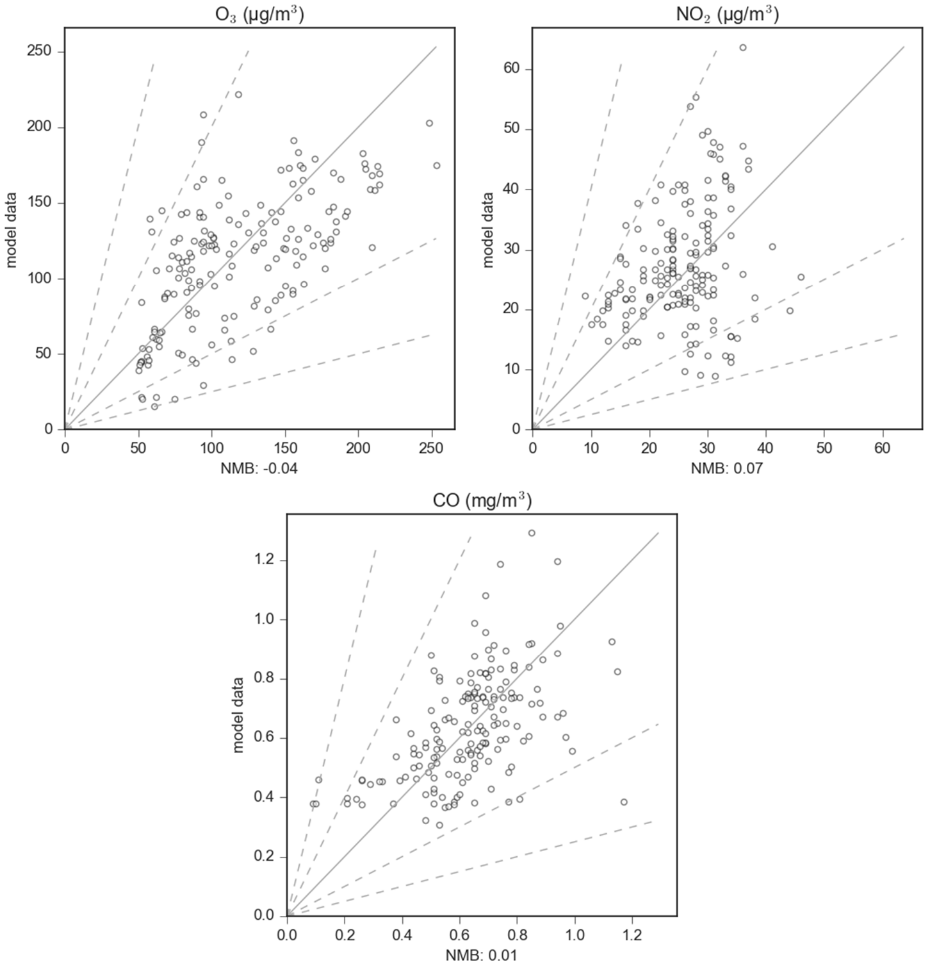

Fig.S4 Scatter plot of O_3, NO_2 and CO concentration in the study area. The model data are resampled into a time resolution of 1 hour to match the observation data. (NMB: Normalized mean bias)

An observation of pollutants CO and NO_2 at the roadside was also done during 27-29 July, 2018. The observation started from 9:30 to 12:00 in the morning, and 14:30 to 17:00 in the afternoon. To avoid the errors during the starting phase of the monitor, the first hour data was discarded. The observation data was resampled into a time resolution of 5 min to be in line with the model data. The results are shown in Fig.S5-6. It can be seen that generally the modelled data is in good correlation with the roadside monitoring data, even though sometimes the roadside data has a higher value which could be due to the roadside vehicle exhaust.

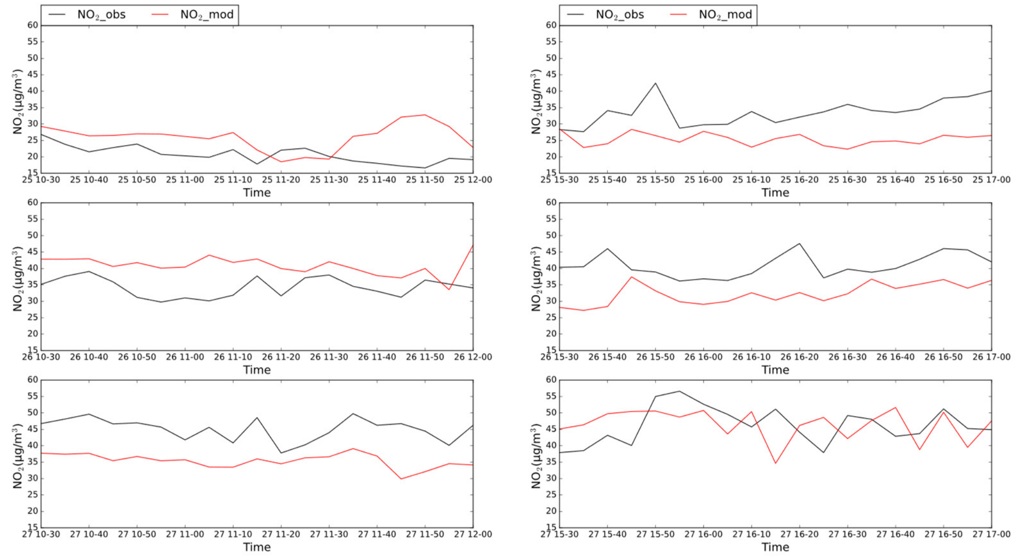

Fig.S5 The concentration of NO_2 from observation data and modelled data. The red line is the modelled data while the black line is the observation data. Time resolution is 5min.

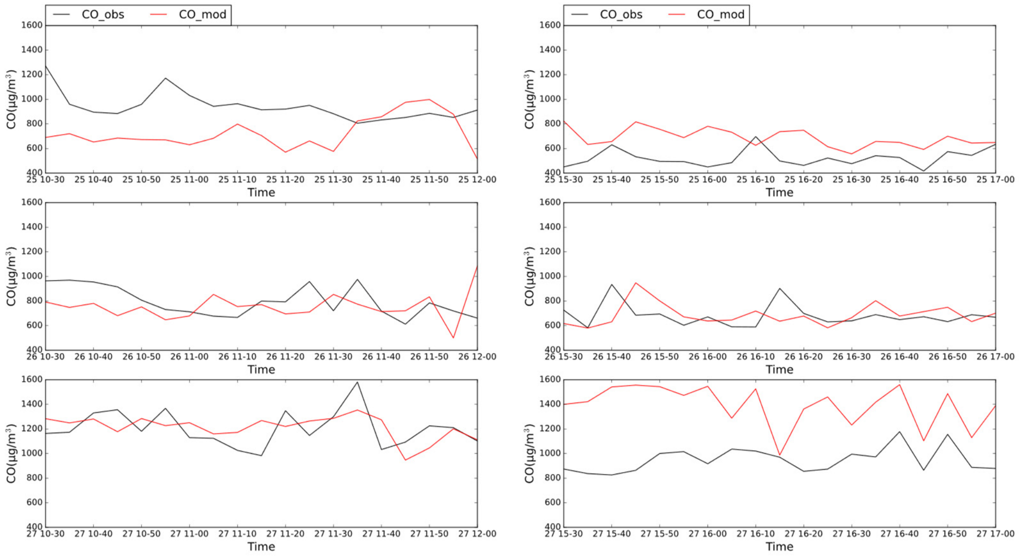

Fig.S6 Same as Fig.S5, but for CO.

An additional model run is also conducted in an area of the same size as the study area (the "test area", as called in the manuscript). The national site 2 is located in the test area and therefore the validation between model results and the national site data are conducted. The time series plot and the scatter plot are shown as Fig.S7-8.

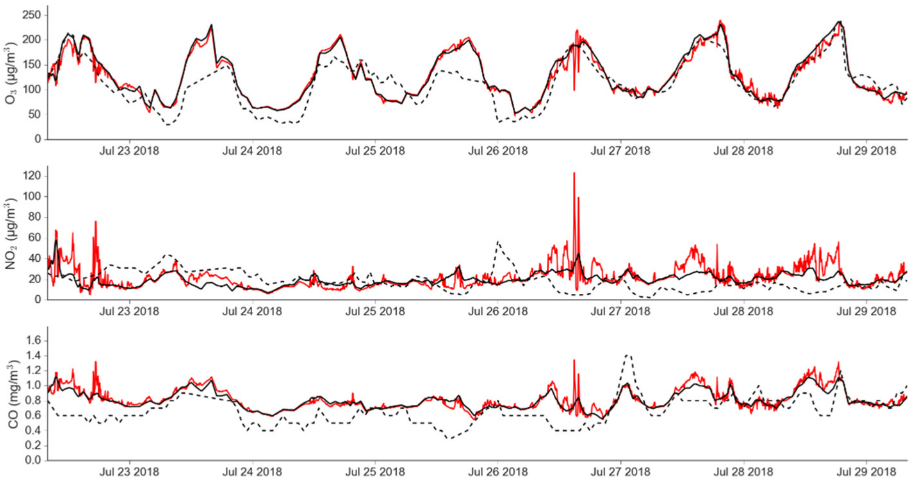

Fig. S7 Time series of the observed and modelled O_3, NO_2 and CO concentrations from 22 July to 29 July 2018 over the test area. The red line shows the model data at the grid where the national site is located, and the black solid line is the spatially averaged model data. The black dashed line is the data from the national site.

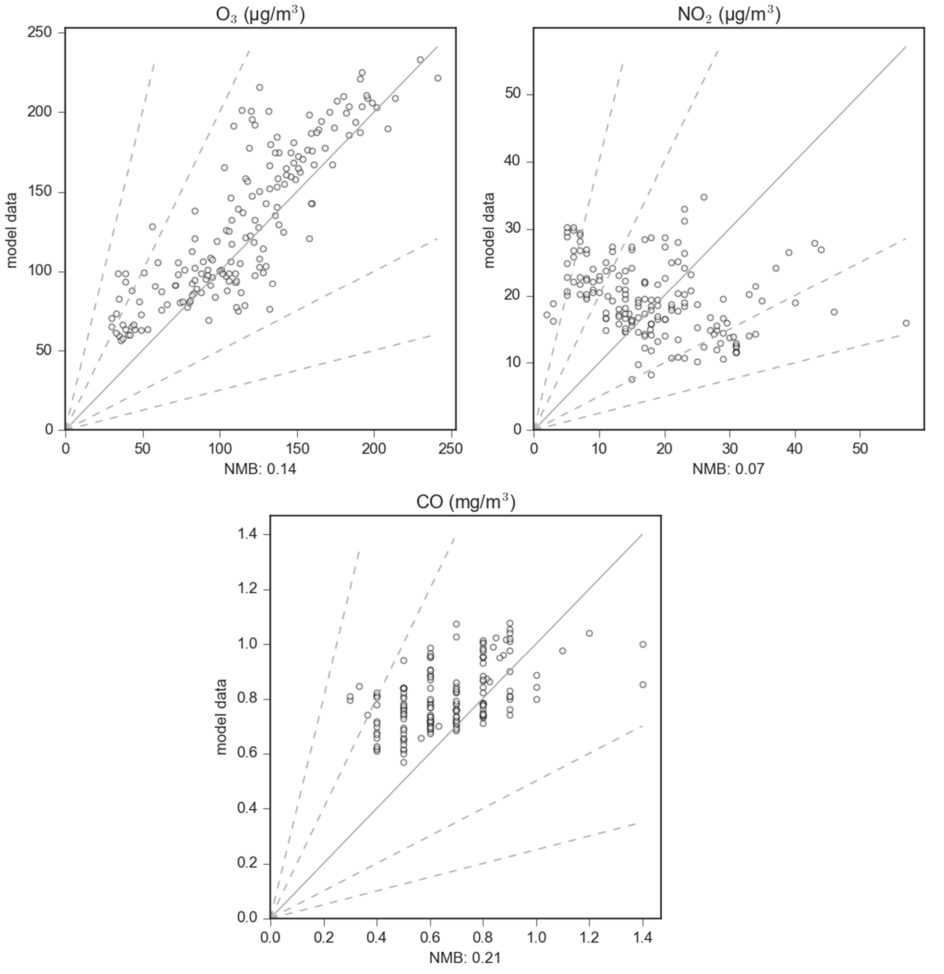

Fig. S8 Same as Fig. S4, but between the model results of the test area and the national site data.

S4. Air pollution distribution patterns in the studied city block

Spatial distribution of O_3, NO_2 and CO

Fig. S9 shows the three-dimensional view of spatial distribution of the key air pollutants (i.e., NO_2, CO, and O_3) over the studied city block in Baoding city. For traffic emitted pollutants, i.e., NO_2 and CO, high concentrations occur mainly along the streets, where traffic emissions are the dominant source for these air pollutants. Over the non-road areas (defined as the areas other than the main streets, i.e., without traffic emissions, please refer to Fig. S2(a) for the traffic emission distribution), due to spatial dilution during the transport as well as the blocking effects of the buildings, the concentrations of these traffic relevant pollutants are significantly reduced. On the contrary, O_3 shows a completely different spatial pattern, where the concentrations are lower in the road areas and are higher over the non-road areas. This distinct distribution pattern of O_3 is determined by multiple factors, such as the NO_X titration originating from traffic emissions, the background O_3 concentration and the wind field.

In the vertical direction, the pollutant concentrations show quick decrease with the height increasing (Fig. S11-S13). In the ground layer, the patterns are largely determined by emissions

and the hindrance of building groups; while in the higher layers, with lower effects from emission and the decreasing number of tall buildings, the pollutants tend to be more uniformly distributed, and are mainly dominated by wind fields and background transport.

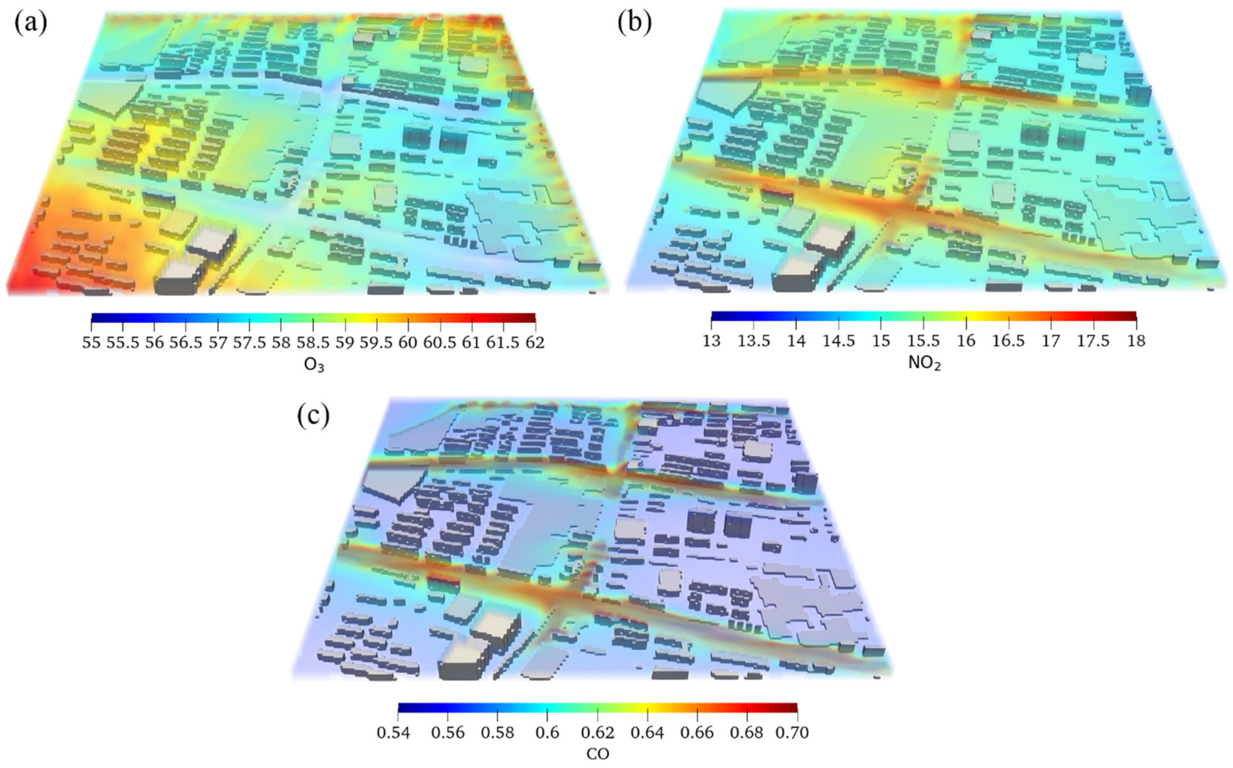

Fig. S9 Three-dimensional distributions of the simulated air pollutants: (a) O_3 (ppb), (b) NO_2 (ppb) and (c) CO (ppm) in the 1km × 1km × 30m city block in the downtown area of Baoding, China, averaged over the period July 22-29, 2018. The colours indicate the concentrations of different pollutants.

EOF analysis of the air pollution distribution

To evaluate the spatiotemporal pattern of the pollutant distribution in the city block, the EOF analysis was performed based on the model results. Fig. S10 shows the first 3 principle mode of the four pollutants in the ground level. For all the pollutants, EOF mode 1 constitutes more than 70% of the variance. The EOF mode 1 has similar spatial distributions for all pollutants except O_3, which is due to the fact that O_3 is not a traffic-emitted pollutant. The patterns of NO_2 and CO are relatively uniform, with only slight difference between main road areas and other areas. The pattern of O_3 is also uniform, except for the higher value on the southeast boundary, which is determined by boundary conditions. Note that the basis function of the first EOF has negative value for all pollutants, which indicates a consistent trend over the whole block. Meanwhile, the temporal pattern of EOF1 (please refer to Fig. S14-S16 in the supporting information) shows clear diurnal cycles. Therefore, the EOF mode 1 can be interpreted as the variability of air pollutants attributing to diurnal cycles and boundary transport.

EOF mode 2 constitutes about 5% of the variance. The spatial patterns of EOF2 are very similar to the averaged spatial distribution of ground-level air pollutants (Fig. S9). The values are

positive inside street canyons but negative over the non-road areas, except for O_3, whose pattern is strongly affected by boundary transport. Since the pollution concentration of value is a product of the spatial mode value and the time series value, the mode 2 pollution component has positive values during the day and negative values during the night over road areas. Therefore, EOF2 is largely related to the dispersion of traffic emissions, which are determined by road emissions, wind field and urban morphology.

EOF mode 3 contributes only approximately 1%-2% of the total variance for all pollutants, and their spatial patterns are all different from each other. The EOF mode 3 of O_3 also shows regional difference between the east part and the west part of the block, which is another effect from the southeast boundary. An interesting similarity exists between the EOF3 of NO_2 and the EOF2 of O_3, showing the important boundary transport from the southwest side of the block for the two pollutants. We can also conclude that NO_2 is much more affected by traffic emission as this EOF mode contribute less variance compared to the mode of O_3. The EOF3 of CO, however, show few signs of boundary effects. In this EOF mode, the main difference lies between different parts of the main streets, and the non-road areas have values very close to 0. Therefore, the third mode of CO can be closely related to the accumulation of CO in different parts of the street canyons.

In the middle layer (z=12.5 m, please refer to Fig. S17), more distinct distribution loading patterns are observed. Fewer buildings reach this height, and the blocking effect of buildings on air flow is much weaker than that in the ground layer. As a result, the spatial patterns of EOF1 show a smooth transition between road and non-road areas. In addition, a much smaller value of EOF2 (2%) also indicate that the direct influence of traffic emissions to this level is greatly reduced. Therefore, these features of EOF1 and EOF2 indicate that at this height, the concentrations of most pollutants are mainly controlled by diurnal variability and boundary transport. In regard to the top layer (z=27.5m, please refer to Fig. S18), the air flow is almost unblocked by buildings and the influence from traffic emissions is trivial. Therefore, air pollutants above 20m are mainly determined by the wind flow and background mixing.

Overall, the spatial patterns of EOF2 are generally in line with the spatial distribution of mean concentrations of different air pollutants, which means that traffic emissions and urban morphology do play an important role in determining pollution distribution at the ground level, and directly affect human exposure to traffic emissions. Although the eigenvalues of EOF1 are the largest ones for most air pollutants, they are mainly contributed by diurnal cycles and background transport and focus more on the general pollution level than the detailed pollution distribution, and are therefore less relevant to the evaluation of chronic human exposure and amelioration of local urban morphology.

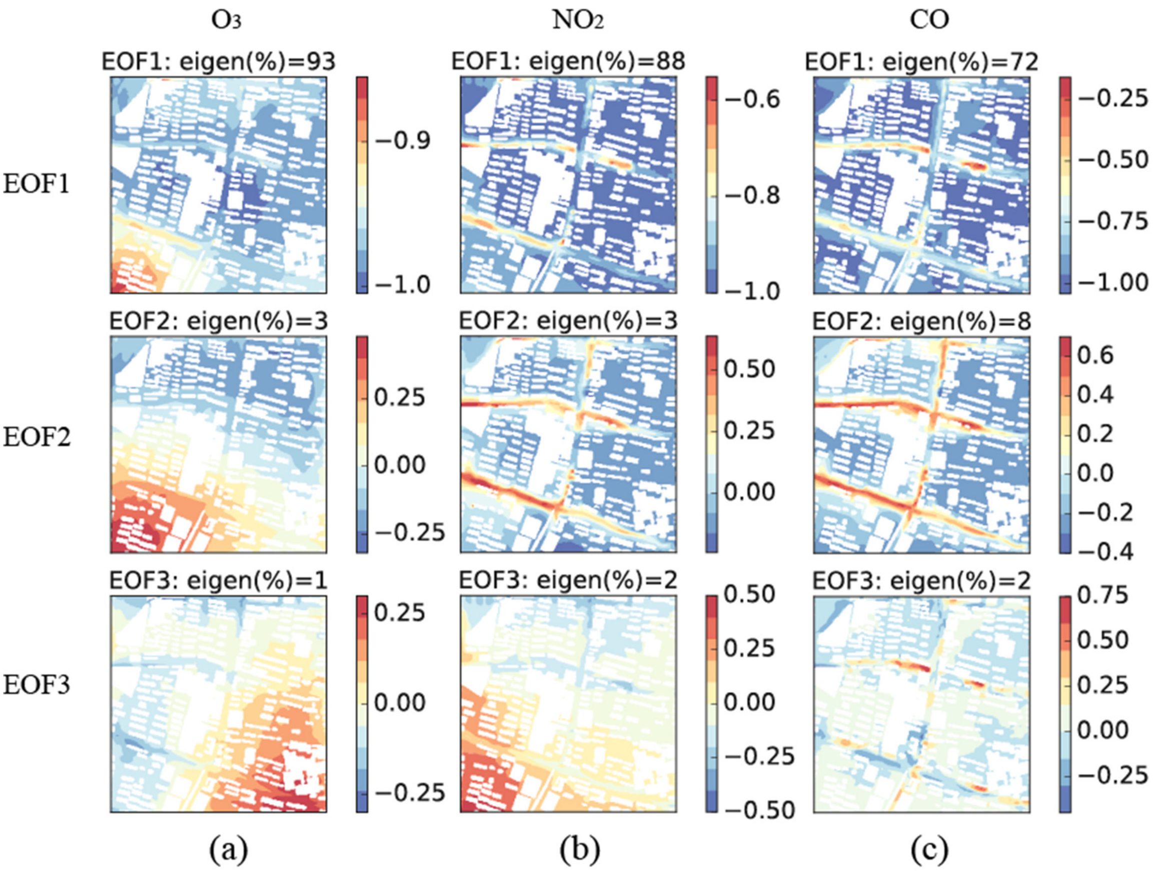

Fig. S10 The spatial modes of EOF1 to EOF3 on the ground level (z=2.5 m) of (a) O_3; (b) NO_2; (c) CO. The eigenvalues of the corresponding EOF patterns are also shown.

S5. Other supplementary materials

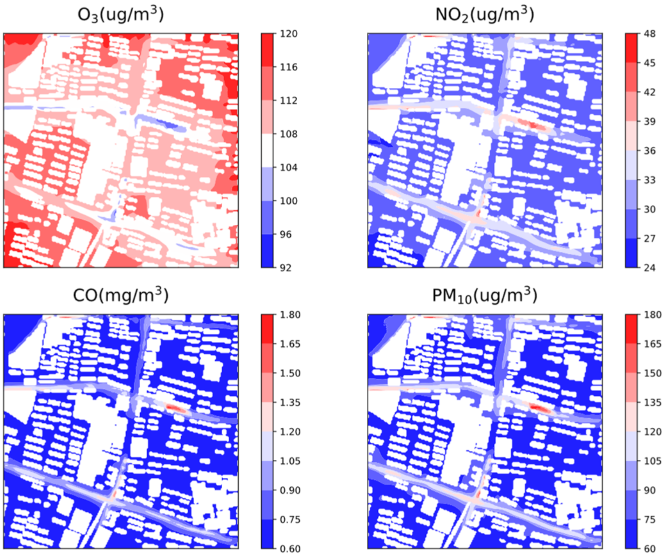

Fig.S11 The time averaged pollution concentration for O_3, NO_2, CO and PM_{10}, at the height z=2.5m.

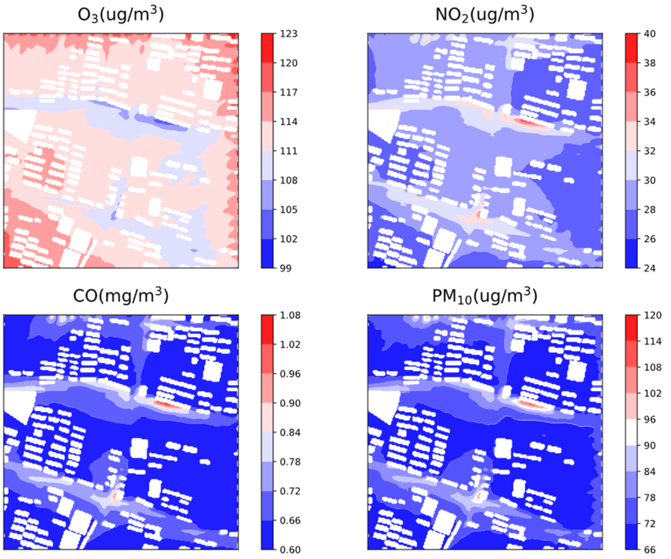

Fig.S12 Same as Fig.S11, but for z=12.5m.

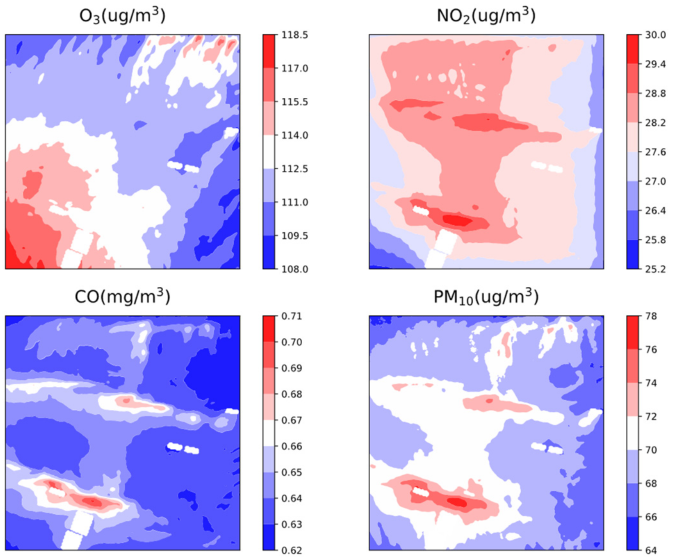

Fig.S13 Same as Fig.S11, but for z=27.5m.

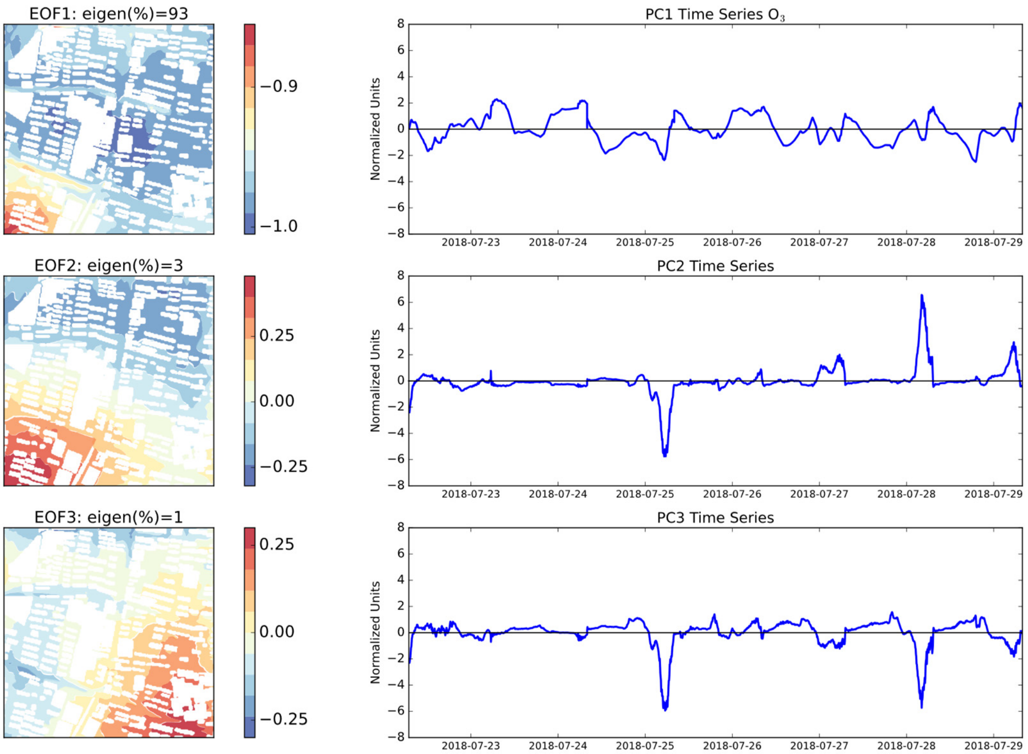

Fig.S14 EOF correlation result of O_3 at z=2.5m. The figures on the left are the spatial patterns while those on the right are corresponding time series.

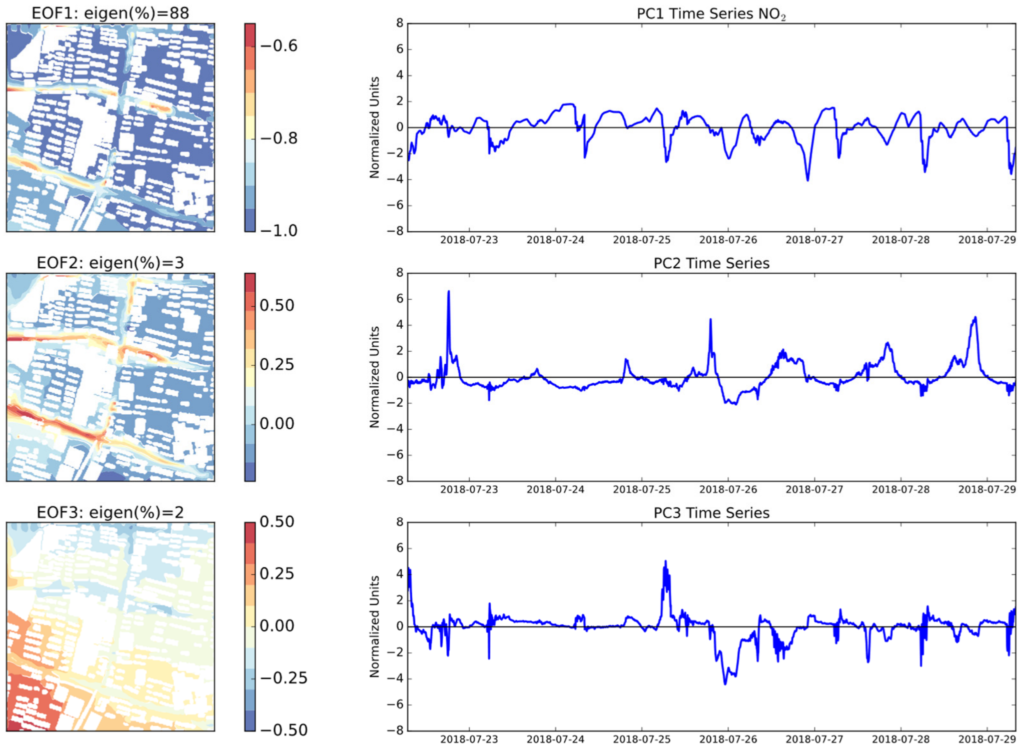

Fig.S15 Same as Fig.S14, but for NO_2.

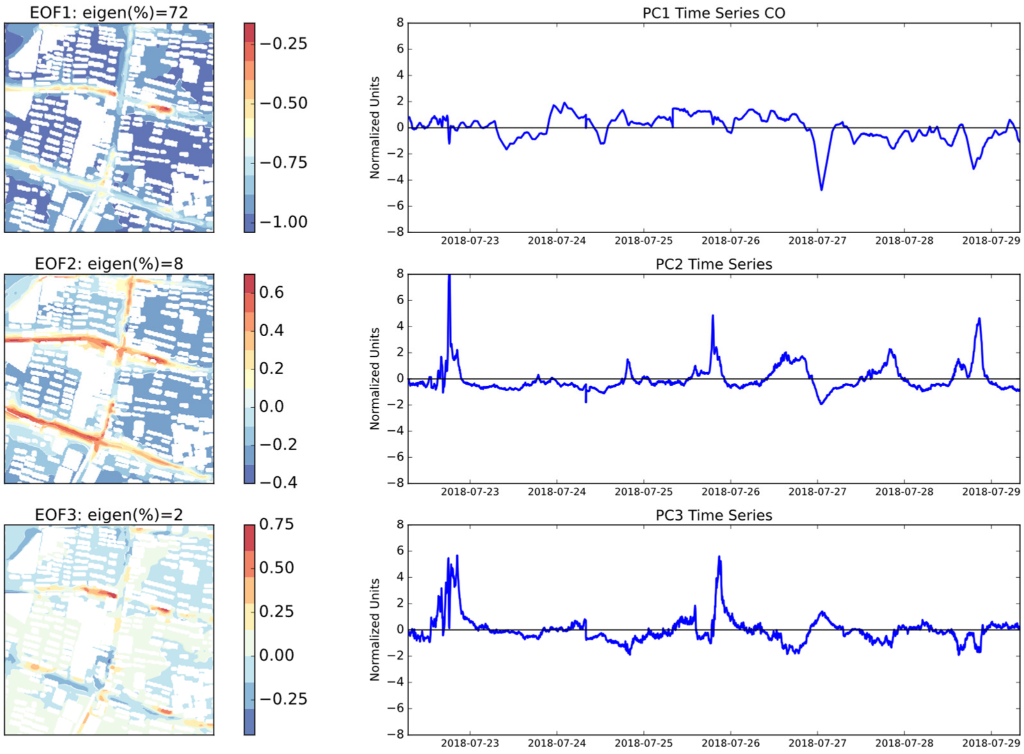

Fig.S16 Same as Fig.S14, but for CO.

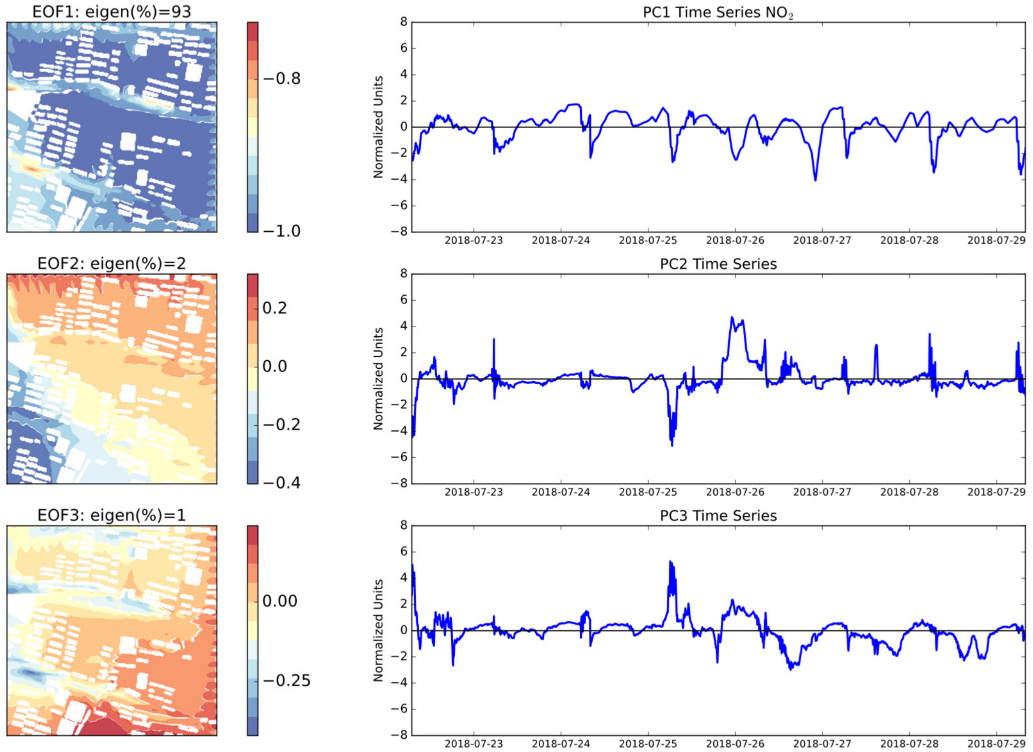

Fig.S17 EOF correlation result of NO_2 at z=12.5m. The figures on the left are the spatial patterns while those on the right are corresponding time series.

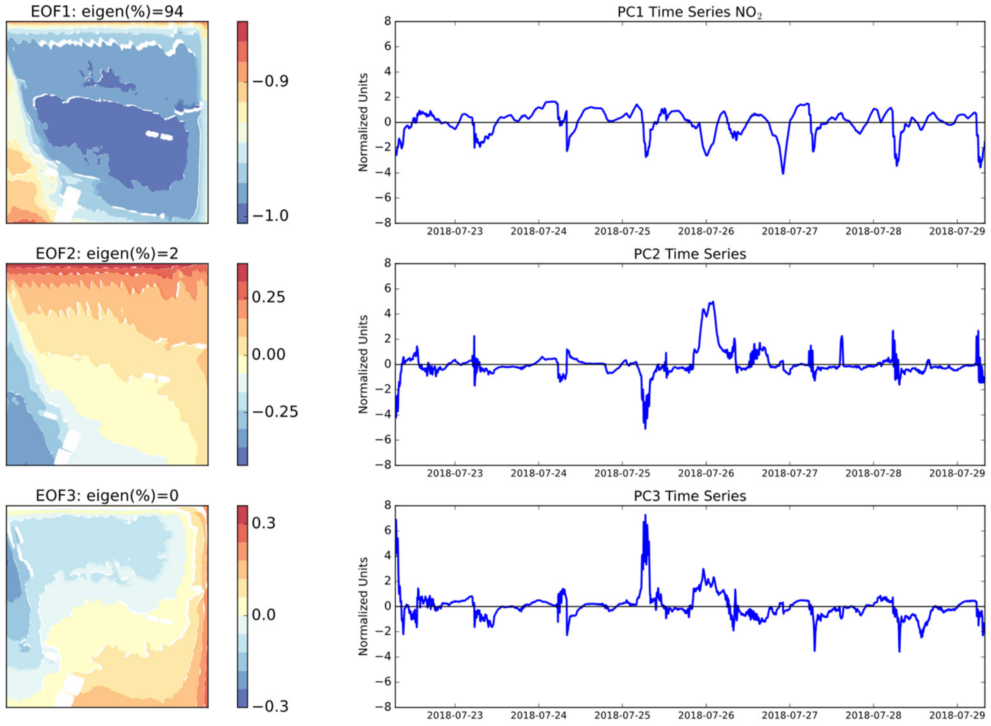

Fig.S18 EOF correlation result of NO_2 at z=27.5m. The figures on the left are the spatial patterns while those on the right are corresponding time series.

Table S2 Correlation coefficients between the pollutants (i.e., NO_2, O_3, CO) and eight influencing factors individually in road and non-road areas at the ground-level

Pollutant	NO_2		O_3		CO	
	road	non-road	road	non-road	road	non-road
Emission	0.79^{**}	/	-0.62^{**}	/	0.82^{**}	/
D_r	/	-0.55^{**}	/	0.41^{**}	/	-0.41^{**}
Aspect ratio (H/W)	0.30^{**}	/	-0.18^{**}	/	0.41^{**}	/
Asymmetry (H1/H2)	0.37^{**}	/	-0.28^{**}	/	0.50^{**}	/
BCR	0.01	0.12^{**}	0.05^{**}	-0.07^{**}	0.02	0.05^{**}
Rugosity	-0.11^{**}	-0.06^{**}	0.27^{**}	0.02^{**}	-0.06^{**}	-0.06^{**}
Occlusivity	-0.03^{**}	0.11^{**}	0.06^{**}	-0.04^{**}	-0.02^{*}	0.09^{**}
Porosity	0.11^{**}	0.05^{**}	-0.27^{**}	-0.01	0.05^{**}	0.07^{**}

1. The values in the table represent the coefficients of the independent variables individually with the pollutant in both road and non-road areas; "/" indicates that this parameter is not considered in the regression model.

2. *: $p<0.05$; **: $p<0.01$.

3. The units of the different pollutants are: NO_2 ($\mu g/m^3$), O_3 ($\mu g/m^3$) and CO (mg/m^3).

Table S3 Correlation coefficients between the standardized pollutants concentration (i.e., NO_2, O_3, CO) and standardized influencing factors individually in road and non-road areas at the ground-level

Pollutant	NO_2 ($\mu g{\cdot}m^{-3}$)	O_3 ($\mu g{\cdot}m^{-3}$)	CO ($mg{\cdot}m^{-3}$)
Emission	0.45^{**}	-0.36^{**}	0.51^{**}
DR (m) [1]	-0.17^{**}	0.18^{**}	-0.08^{**}
Aspect ratio (H/W)	0.07^{**}	0.05^{**}	0.07^{**}
Asymmetry (H1/H2)	0.08^{**}	-0.09^{**}	0.14^{**}
BCR	0.11^{**}	-0.14^{**}	0.07^{**}
Rugosity	-0.04^{*}	0.07^{**}	-0.01
Occlusivity	-0.04^{**}	0.06^{**}	-0.03^{**}
Porosity	0.03^{**}	-0.04^{**}	0.02^{**}

1. The values in the table represent the standardized coefficients of the independent variables.

2. *: $p<0.05$; **: $p<0.01$.

Table S4 Correlation between the UMPI and the standardized pollution concentration (i.e., NO_2, O_3, CO) in the ground-level over the test area

	NO_2	O_3	CO
r	0.58	0.57	0.64
k	0.007	-0.009	0.010

Table S5 Standard errors for results in Table 2

Pollutant	NO_2 ($\mu g{\cdot}m^{-3}$)	O_3 ($\mu g{\cdot}m^{-3}$)	CO ($mg{\cdot}m^{-3}$)
Emission	1.6e-7	2.6e-7	1.5e-5
DR (m) [1]	1.0e-7	1.6e-7	7.7e-6
Aspect ratio (H/W)	8.3e-5	1.3e-4	0.006

Asymmetry (H1/H2)	5.0e-5	7.8e-5	0.004
BCR	7.3e-5	1.1e-4	0.006
Rugosity	6.3e-6	9.8e-6	4.8e-4
Occlusivity	5.8e-5	9.1e-5	0.004
Porosity	7.8e-5	1.2e-4	0.006

Table S6 Standard errors for results in Table 3

Pollutant	NO_2 (μg·m^{-3})	O_3 (μg·m^{-3})	CO (mg·m^{-3})
DR (m) [1]	1.1e-7	1.5e-7	9.4e-6
Aspect ratio (H/W)	1.0e-4	1.4e-4	0.009
Asymmetry (H1/H2)	6.2e-5	8.3e-5	0.005
BCR	9.3e-5	1.2e-4	0.008
Rugosity	8.0e-6	1.0e-5	0.001
Occlusivity	7.4e-5	1.0e-4	0.006
Porosity	1.0e-4	1.3e-4	0.008

Table S7 Standard errors for results in Table 4

Pollutant	NO_2		O_3		CO	
	road	non-road	road	non-road	road	non-road
Emission	0.018	/	0.020	/	0.57**	/
DR (m)	/	0.003	/	0.004	/	-0.07**
Aspect ratio (H/W)	0.017	/	0.019	/	0.36**	/
Asymmetry (H1/H2)	0.015	/	0.016	/	0.23**	/
BCR	0.025	0.005	0.028	0.006	0.02	0.01**
Rugosity	0.064	0.008	0.070	0.009	-0.15**	-0.02**
Occlusivity	0.024	0.004	0.027	0.005	-0.03*	0.02**
Porosity	0.022	0.003	0.023	0.004	0.05**	0.01**

Reference:

1. Adolphe, L.J.E. A simplified model of urban morphology: Application to an analysis of the environmental performance of cities. *Environ. Plan. B Plan. Des.* **2001**, *28*, 183–200.
2. Yang, J.; Shi, B.; Zheng, Y.; Shi, Y.; Xia, G. Urban form and air pollution disperse: Key indexes and mitigation strategies. *Sustain. Cities Soc.* **2019**, *57*, 101955.
3. Vittinghoff, E.; Gliddon, D.V.; Shiboski, S.C.; McCulloch, C.E. *Regression Methods in Biostatistics*; Springer: New York, NY, USA, 2005.
